# Stiff Composite Cylinders for Extremely Expandable Structures

**DOI:** 10.1038/s41598-019-51529-7

**Published:** 2019-11-04

**Authors:** Arthur Schlothauer, Paolo Ermanni

**Affiliations:** ETH Zürich, Laboratory of Composite Materials and Adaptive Structures, Zürich, 8092 Switzerland

**Keywords:** Composites, Mechanical engineering

## Abstract

The realization of concurrently largely expandable and selectively rigid structures poses a fundamental challenge in modern engineering and materials research. Radially expanding structures in particular are known to require a high degree of deformability to achieve considerable dimension change, which restrains achievable stiffness in the direction of expanding motion. Mechanically hinged or plastically deformable wire-mesh structures and pressurized soft materials are known to achieve large expansion ratios, however often lack stiffness and require complex actuation. Cardiovascular or drug delivery implants are one example which can benefit from a largely expandable architecture that is simple in geometry and intrinsically stiff. Continuous shell cylinders offer a solution with these properties. However, no designs exist that achieve large expansion ratios in such shells when utilizing materials which can provide considerable stiffness. We introduce a new design paradigm for expanding continuous shells that overcomes intrinsic limitations such as poor deformability, insufficient stiffness and brittle behaviour by exploiting purely elastic deformation for self-expandable and ultra-thin polymer composite cylinders. By utilizing shell-foldability coupled with exploitation of elastic instabilities, we create continuous cylinders that can change their diameter by more than 2.5 times, which are stiff enough to stretch a confining vessel with their elastic energy. Based on folding experiments and analytical models we predict feasible radial expansion ratios, currently unmatched by comparable cylindrical structures. To emphasize the potential as a future concept for novel simple and durable expanding implants, we demonstrate the functionality on a to-scale prototype in packaging and expansion and predict feasible constellations of deployment environments.

## Introduction

Expandability and stiffness in cylindrical or spherical structures are two fundamentally conflicting properties. Considerable diameter changes in continuous cylinders or spheres require large amounts of circumferential strain, which given a higher modulus material results in a significant amount of actuation forces. Existing solutions for expandable systems avoid stiff materials and couple soft structures with pressurized actuation^1–4^, chemical reactions^5^ or shape memory effects^6,7^. Structures where soft materials can not meet stiffness requirements, independent of external actuation, have to deviate from the desirable continuous shells to hinged lattice-^8,9^ or wire-like architectures^10^, which achieve considerable amount of shape change through kinematics^11^, plastic deformation^12^ or superelasticity^10^.

Especially at a smaller scale, deviations from a cylindrical continuous shell can result in notable drawbacks. A recent example are transcatheter aortic valve replacement (TAVR) stents for the treatment of calcified heart valve leaflets inside the human body. A TAVR-device is crimped to a diameter of around 10 mm and then non-invasively implanted using a catheter. Once it has reached the aortic valve opening, the device is expanded to more than 2.5 times of its packaged diameter to permanently fixate the prosthesis at its intended spot. The requirements for the expanding structures demand both radial stiffness, to create sufficient fixation forces in the artery, and extensive radial deformability^13^. Because of the wire-like nature, state-of-the-art devices are currently composed of a soft prosthesis replacing the native valve and a metallic wire-mesh delivery device^13,14^ which have to be connected together using a sub-optimal hand-suture process^15,16^. This negatively influences durability and implant cost. The utilization of thin shell structures for the delivery device is therefore not only highly desirable from a structural standpoint but its continuous surface area also provides the pre-requisite for optimized interfaces^17^ between the soft prosthesis and the stiff support.

Because of the distinct structural and practical advantages of cylindrical shells, we propose a new paradigm for largely expandable structures overcoming the drawbacks associated with known radially expandable solutions. Namely, we combine instability inspired folding patterns with highly anisotropic material properties to achieve counter intuitive functionality of permanent dilation despite using basic cylindrical geometries and linear elastic materials. Applying this idea to the mechanical boundary conditions of TAVR devices, we show that extreme expansion ratios, even under the influence of a smaller confining vessel like a human artery, do not require shape memory effects, plastic deformation or external driving forces and can be achieved with instability-enhanced functionality^18,19^ in continuous polymer composite cylinders. We demonstrate the functionality using thin-ply laminated carbon fiber reinforced epoxy cylinders.

## Concept of Radial Expansion

**Figure 1:**
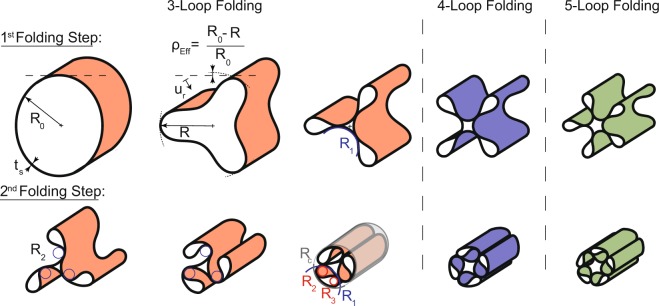
Schematic introducing the folding pattern and essential geometric parameters of the linear elastic continuous shell structure. The folding process is described through the radial folding distance, *u*_*r*_, (non-dimensionalized with respect to the shells initial circular radius, ${\delta }_{fold}={u}_{r}/{R}_{0}$), and the continuously changing radius, *R*, (the radius of the circle formed by the lobe tips). The change of *R* with respect to its initial diameter can be described with the non-dimensional parameter, ${\rho }_{eff}$, also a measure for packaging efficiency. The fully packaged state can be parametrized purely through geometry using the structures thickness, *t*_*s*_, and four tangential radii *R*_1_, *R*_2_, the lobe tip radius *R*_3_, and *R*_*c*_.

The utilization of thin composite cylinders allows for the combination of high modulus and locally tunable bending deformability. The significant hoop stiffness associated with the stiff material and the undeformed cylindrical geometry provide great dimensional stability against pressure loads. When deformed in the right way, the stored strain energy of the elastic material can be used for autonomous expansion (as used in slender space trusses or booms^20,21^) to recover the cylindrical shape and act as a pressure load stretching an undersized external confinement like an human artery. A key factor to realize folded shapes that induce a radial autonomous expansion through strain energy recovery are elastic instabilities. Consequently, we seek to induce instabilities in the cylindrical shell inspired by the higher order buckling modes of a cylinder under radial pressure^22^ (Fig. 1, first row). We chose these modes because their deformation pattern is causing a reduction in diameter and hence a radial expansion after releasing the structure. After inducing the instabilities, the lobed structures allow to further fold the shell. Drastic reduction of diameter can then be achieved by chirally twisting lobes around eccentric axes in a clock- or counter-clockwise direction (Fig. 1, second row). The folding concludes when adjacent lobes come into contact, forming a chiral structure with radius, *R*_*c*_, which can be inserted into a cylindrical sleeve (or a catheter). Inside its sleeve, the structure is self-equilibrating and keeps its chiral shape. Once released from its sleeve, the shell autonomously expands according to its folding pattern.

## Materials and Methods

### Manufacturing of ultra-thin CFRP cylindrical shells

The ultra-thin cylindrical shells were manufactured using 20 g m^−2^ unidirectional carbon fiber spread tow prepreg (T700SC fiber (Toray) and TP402 resin manufactured by North Thin Ply Technologies). The plies were wound around a cylindrical steel mandrel (29 mm diameter) and fully impregnated under 80 °C for 30 min following a complete cure at 125 °C and 0.6 MPa external pressure for 2 h. The cure resulted in structures of 122 μm thickness on average (obtained by optical microscopy with a Keyence VHX6000 digital microscope), resulting in an average fiber volume content of 55%. Larger scale samples were manufactured accordingly with increased diameter steel mandrels. After cure, the samples were cut into lengths of 15 mm and the edges sanded to remove remaining cracks and imperfections from cutting.

### Folding of the ultra-thin cylinders

**Figure 2:**
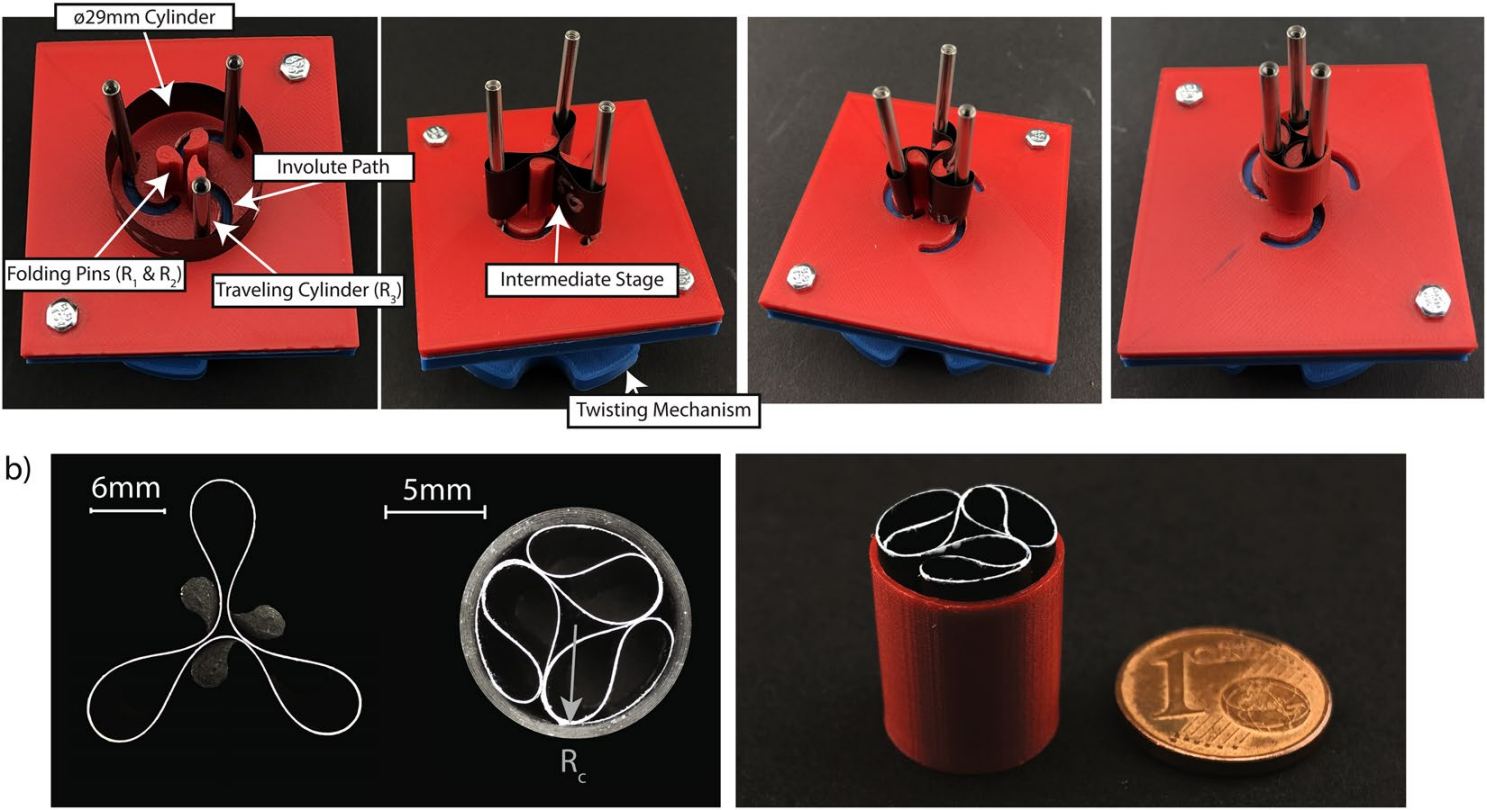
(**a**) Folding sequence with 3D-printed fixtures prescribing the folding radii on a 122 μm thick carbon-fiber epoxy cylinder with an initial diameter of 29 mm. (**b**) Photographs of the intermediate and final folding stage of the folded cylinder (left). And the diameter of the completely folded structure which was removed from the folding jig, compared to a one-cent coin (right).

For the folding of the cylindrical shells 3D-printed folding rigs were designed (Fig. 2 and Supplementary Fig. S1). The folding rigs contain *N* elevated pins (according to buckling mode) prescribing the desired folding radii *R*_1_ when inserting the structure (by hand), as well as the radius *R*_2_ when twisting the structure around the pin. The twisting is achieved by *N* steel cylinders with radius *R*_3_ travelling on centro-symmetric involute paths around the center of the rig. The outer edge of the involute path was defined according to the lobe tip position of the first folding stage of the cylinder. Except for the steel cylinders, all other parts were 3D-printed from Polylactic acid with an Ultimaker 2+. The folding concludes by pulling a thin 3D-printed sleeve with crimped radius *R*_*c*_ over the packaged structure that constrains the fiber reinforced cylinder. After folding, the folding rig can be removed and the structure self-equilibrates inside its sleeve.

### Estimation of sustainable bending radii of the prototype material

**Figure 3:**
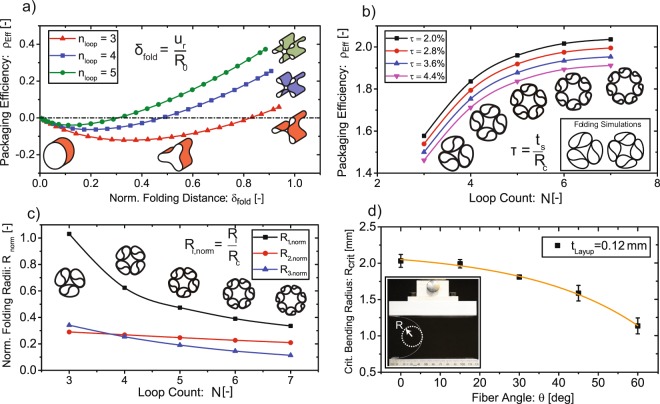
(**a**) Numerical simulation of the evolution of the packaging efficiency during lobe formation showing positive values for lobe counts ≥3. Furthermore, the maximum achievable expansion increases with increasing lobe count. (**b**) Prediction of the maximum packaging efficiency of the second folding step as a function of the number of lobes derived from a circle packaging problem. Efficiency increases initially with lobe count, stagnating at higher lobe counts due to unused area in the middle of the chiral structure. Furthermore, a comparison between numerical simulations and the geometric model is shown. (**c**) Normalized radii *R*_*i*=1,2,3_ (Shown in Fig. 1 and SI) resulting in optimal geometric packaging efficiency of a 120 μm thick structure, showing a reduction of optimum packaging radii with increasing lobe count. (**d**) Minimum bending radius of balanced and symmetric carbon fiber epoxy layups as a function of fiber angle. 0° is in the direction of the curvature and error bars show the standard deviation of test samples (five samples per fiber angle). This demonstrates the outstanding flexibility of ultra-thin layups and the possibility to tune the structures by varying the fiber angle according to their folding schemes.

To design layups which sustain the bending radii of the folding pattern, platen tests^23,24^ were performed on symmetric and balanced 4-ply layups ([0]_4_, [15/−15]_*S*_, [30/−30]_*S*_, [45/−45]_*S*_ and [60/−60]_*S*_, with 0-degree following the bending curvature of the specimen) of 80 μm thickness and a fiber volume content of 55% of the prototype material. The final curvature at failure of the specimen was captured by a high resolution camera (Nikon D5300 with lens NIKKOR 105 mm 1:2.8 G) and evaluated using circular hough transformation in Matlab (Fig. 3d). Using the purely geometric relation of strain $\varepsilon$, curvature $\kappa$ and distance from the neutral axis, *z*, the maximum bending strains can be approximated with:

$${\varepsilon }_{{\max }}={\kappa }_{{\max }}\cdot \frac{{t}_{s}}{2}$$

The critical bending radius hence results from the layup thickness and the inverse of $\kappa$. For the material test, each layup included a sample size of five coupons with rectangular dimensions of 120 mm × 40 mm. Thicker layups can be extrapolated from the test results if their thickness does not vary more than twice the tested thickness, since size effects might result in a change of failure mechanism.

### Numerical simulations of folding and deployment

To predict folded geometry, resulting forces and feasible deployment environments, non-linear numerical simulation of the folding and deployment were created in ABAQUS/CAE and solved with ABAQUS/Standard. The simulation model was based on S4R shell elements with linear elastic orthotropic material properties of *E*_11_ = 139 GPa, *E*_22_ = 8.72 GPa, *G*_12_ = 3.39 GPa and *ν*_12_ = 0.23. The structure was folded with rigid body pinching elements with the shape of the folding rig pins assembled with the radius *R*_1_ and *R*_2_. Contact was modelled as hard surface contact with friction between folding pins (friction coefficient ${\mu }_{1}=0.8$) and structural self-contact with ${\mu }_{2}=0.1$. The layup of [60°/−60°/0°]_*Sym*_ was modelled with ply thicknesses of 20 μm. Radial displacement of the lobe tip were extracted as well as the folding distance and radial forces obtained from the rigid body reaction forces. The folding distance of the first stage is a function of lobe count *N* and followed the equation:

$${\delta }_{fold,max}=1+\frac{1}{{R}_{0}}\cdot ({R}_{1}-\frac{{R}_{1}+2{t}_{s}}{sin(\frac{\pi }{N})})$$

The second stage follows a rotation of the lobe tips around the pinching bodies until enclosed by a rigid body cylinder simulating the outer constraint.

For the simulation of the deployment process in a confined environment, the structure was folded into its lobed shape to be consequently released in a confining vessel. After folding, the structure of initial radius *R*_0_ was moved into a linear elastic vessel of radius *R*_*V*_ modelled with S4R elements of stiffness *E*_*V*_, thickness *t*_*V*_ = 2.2 mm, Poisson’s ratio *ν*_*V*_ = 0.45 and a length 5 times larger than the structure length to avoid edge effects. The vessel was clamped at both ends to remove rigid body motion. The pinching bodies which folded the structure were released (no contact between pinching bodies and vessel) in a quasi-static manner and the developing reaction forces the rigid bodies extracted while the structure makes contact with the vessel.

### Deployment tests in confined vessels

To verify the feasibility of the folding and expansion concept, deployment tests were carried out using an scaled-up version of the expanding structure (diameter 51 mm, length 25 mm). The confining vessel was manufactured by casting Altropol Protosil RTV 245 Silicone (Shore A40, ≈*E*_*V*_ = 1.7 MPa) into a 3D-printed Polylactic Acid (PLA) mold resulting in a vessel thickness of *t*_*V*_ = 3.5 mm and radius of 49 mm. The relation between structure bending stiffness and vessel hoop stiffness was tuned to represent the scale 1:1 application (Supplementary Information). The silicone vessel was mounted onto a PLA fixture from both sides and fixed at the edges using ethyl cyanoacrylate to restrict the movement of the vessel edges. The deployment test setup is shown in Supplementary Fig. S3. The structure was folded with the designed folding rig and deployed by pushing it outside the PLA sleeve. Recordings were taking using the high-speed camera Photron FASTCAM Mini UX100 at 8500FPS (Supplementary Movie 1) and post-processed using Photrom FASTCAM Viewer as well as Adobe Photoshop to remove blur and unnecessary background. The structure edges were painted in white color to facilitate contrast. The deployment was accepted as successful, if the structure retained its circular shape in five consecutive trails of three different specimen of same dimensions. It shall be mentioned, that the setup of the deployment test should elaborate a one-time deployment in tissue-like environments and therefore does not investigate influences of fatigue, damage or imperfections which are to be investigated in follow-up studies.

## Results and Discussion

### Packaging efficiency of stiff composite cylinders

The creation of the buckling-inspired lobed structures relies on an equal distribution of N radial loads pinching the structure along the circumference. The pinching motion is concluded when the inner portions of the structure touch each other (Fig. 1, first row). By changing the amount of pinching bodies, one can achieve higher order instability patterns, illustrated as 4- and 5-loop folding. Favourable are the folded shapes for $N\ge 3$ because the enclosing radius, *R*, after concluding the first folding step, is smaller than the unfolded radius *R*_0_. This results in a positive packaging efficiency (${\rho }_{eff}=({R}_{0}-R)/R$, Fig. 3a, Methods) and hence an increase in diameter during expansion. With higher *N*, we can further increase the packaging efficiency in exchange for larger required folding forces^25^.

Following the buckled intermediate state (1st folding step), the chiral fold (2nd folding step) ensures an increase of packaging efficiency by over 100% as can be seen in Fig. 3b. To predict the largest structure that can be packaged into a given diameter and hence shows the highest packaging efficiency, we maximize the arc length of the fully folded shape which is given by the combination of the different folding radii (*R*_1_, *R*_2_ and *R*_3_, seen in Fig. 1). Constrained by a sleeve with radius *R*_*C*_, this leads to a circle packaging problem (For the derivation of the model the reader is referred to the Supplementary Information (SI)). The solution of the geometric optimization is a function of the lobe count and the ratio between desired packaged radius, *R*_*C*_, and shell thickness, *t*_*s*_ (Fig. 3b, SI). With increasing lobe count, the packaging efficiency improves since two folded and rotated lobes can store more arc length than a single larger lobe. The efficiency, however, stagnates at large *N* due to losses in usable area in the centre of the chiral structure, resulting in smaller maximum radial folding distance *δ*_*fold*_ (Fig. 3a). Furthermore, the folded lobe tip radius, *R*_3_, decreases with increasing *N*, imposing curvature requirements the material must withstand. As the curvature and the failure strain are related to each other by the relation ${R}_{{\min }}={t}_{s}/2{\varepsilon }_{{\max }}$, the material should be as thin as possible to increase packaging efficiency. It shall be mentioned, that the fully folded structure slightly deviates from the purely geometrical optimization as a consequence of self-contact and the stored strain energy, which however can be accurately predicted by finite element simulation (Fig. 3b).

### Layup design

At the scale of a TAVI-Implant with an initial shell diameter of 29 mm and wall thickness of 120 μm, optimized efficiency will result in a packaged structure of 11.5 mm, which is comparable to state-of-the-art expandable devices^13^. The platen folding tests of the prototype material allow to choose the fiber angle which provides the maximum stiffness and still allows for folded configurations with 3- or 4 lobes (Fig. 3c). Results show the outstanding flexibility and tunability of the ultra-thin material according to the folding requirements visible by a drastic decrease of bending radius with increasing fiber angle. Ideally, the structure should contain as much circumferentially oriented fibers as possible, since unidirectional flexures loaded in the direction of fibers show the highest ratio of stiffness to critical bending radius resulting from the ability of carbon fibers to recover large strains when loaded in fiber direction^23,26^. However, since purely unidirectional layups also show very low transverse strength, these layups are very prone to premature failure through imperfectly introduced loads during folding. By adding angle-plies sandwiching the circumferential layers this can be mitigated. Furthermore, the overall radial stiffness of the structure can be increased due to the higher strain to ultimate failure of the angle-ply layers (Fig. 3) resulting from a more compliant matrix. The outcome was a 120 μm thick cylinder with a symmetric layup of [60°/−60°/0°]_*S*_ (with 0 degree following the circumference of the cylinder), which was subsequently folded into the structure that can be seen in Fig. 2.

### Expanding the chiral structures

**Figure 4:**
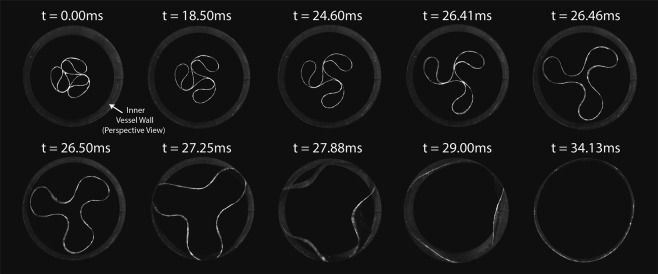
High-speed camera images (8500 fps) of the deployment of a 3-lobed chiral structure inside a confining vessel. A scale 2:1 folded structure is deploying inside a 3.5 mm thick silicone cylinder with a 49.5 mm diameter. The ratio between the structural bending stiffness and vessel hoop stiffness was chosen to be comparable to that of an artificial heart valve stent application (SI).

To demonstrate the feasibility of the expansion, we captured the deployment process of the folded structure with a high-speed camera (Fig. 4, Methods, and Supplementary Movie 1). We observe that upon releasing the folded structure from its sleeve, the stored elastic energy is released, forcing the structure back into its original unloaded state. The deployment takes only 34 ms. The structure retains its centrosymmetric shape until 26.5 ms before the lobe tips make contact with the vessel, allowing simultaneous radial snap-through of the instabilities introduced in the intermediate stage. Following snap-through, the cylindrical vessel is stretched due to the slight oversizing of the expanding structure and hence contact pressure develops.

The folding and unfolding mechanisms are simulated by radially moving pinching bodies in a quasi-static non-linear simulation (Methods). Results deliver the radial reaction forces, *F*_*R*_, and the corresponding radial displacements of the snapping region, *δ*_*fold*_, during snap-through under elastic confinement and can be used to predict limits for successful expansion. A cylindrical shell under sufficient external pressure will collapse into a well defined instability pattern which coincides with the folded structure. Therefore, we can consider the expansion process as the reversal of the buckling instabilities whereas the amount of released energy must be proportional to the critical buckling pressure of the deploying structure, which in the case of a composite cylinder is directly driven by the bending stiffness of the shell in circumferential direction, *D*_22_. Since a confining vessel is opposing the expanding motion, and has to be stretched circumferentially to accommodate the bigger deploying structure (Oversizing $\Omega =({R}_{0}-{R}_{V})/{R}_{V}$), the vessel’s hoop stiffness has to be considered as a restraining factor for successful deployment. The ratio between the critical buckling pressure of the expanding structure and vessel hoop stiffness at constant oversizing can be described through the parameter $\Psi ={D}_{22}/({E}_{V}{t}_{V}{R}_{0}^{2})$ (SI). The dependence of the deployment with respect to parameter $\Psi$ is investigated in the simulation.

**Figure 5:**
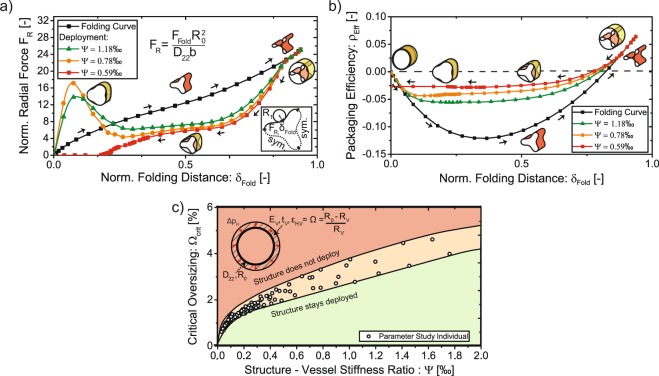
Quasi-static simulation predictions of expansion. Study of the effects of driving parameters on the deployment process. (**a**) Simulation of the normalized reaction forces on the radially moving pinching bodies during folding (black curve, *δ*_*fold*_ increases) and quasi-static expansion (coloured curves, *δ*_*fold*_ decreases). (**b**) The evolution of the effective radius in the simulations described in (**a**) showing the influence of vessel deformability on the corresponding force-displacement behaviour of the snapping region. (**c**) Maximum achievable oversizing of a stiff continuous shell subjected to a radial pressure resulting from the stretching of its confinement as a function of $\Psi$. The results are based on a simplified model predicting the limits on a deployable continuous shell structure (Methods).

While the reaction forces are monotonically increasing during folding, the release of the folded structure in a confined environment shows a typical snap-through behaviour^27^ which is characterized by a negative stiffness region immediately preceding the unloaded configuration (Fig. 5a). By comparing the evolution of the effective radius with the reaction forces (Fig. 5a,b) during folding and deployment, we find that the amplitude of the snap-through gets larger, the less deformable its confining vessel is (small $\Psi$). Considering the deployment process as a reversal of buckling instabilities in a confined cylinder, we can explain the increasing snap through energy by the increase of critical buckling pressure of the confined structure with rising vessel stiffness^28^. Additionally, we find that there is a critical value of $\Psi$, where the structure does not deploy and enters equilibrium with its confining vessel (red curve in Fig. 5a), evident due to a missing snap through and a non-zero effective radius at ${\delta }_{fold}=0$. With decreasing deformability of the vessel, the reaction forces from the deployment will prevent the structure from opening and bifurcate it into the state of a buckled ring under external confinement^29^.

We can estimate whether a given combination of $\Omega$ and $\Psi$ will allow successful deployment by simulating the inverse problem of the expansion process. Namely, an already deployed ring will buckle when the oversizing results in a contact pressure that exceeds its critical buckling pressure with elastically confined boundaries (Fig. 5c). Consequently, if the structure buckles, then it surely will not deploy. For the estimation of the critical buckling pressure in a confined environment, the deployment simulation framework was used. However, instead of folding the structure, the circular shell was directly confined by a soft constraint and loaded under hydrostatic pressure. To enable buckling in the non-linear simulation, imperfections patterns based on the second buckling eigenmode *N* = 3 (corresponding to the folding pattern) were introduced using point loads, deforming the structure with a radial amplitude of 0.5 · *t*_*s*_. The critical buckling point was obtained by scanning the radial displacement of all nodes of the structure and identifying a sudden increase in deformation under linearly increasing hydrostatic pressure. The pressure correlating to the sudden increase was used to estimate the true vessel size *R*_*V*_ that would create the corresponding contact pressures due to stretching using the Lamé-Equations:

$$\Omega \cdot {R}_{V}=\frac{(\frac{1-{\nu }_{V}}{{E}_{V}}){R}_{V}^{2}\cdot {p}_{crit}}{{({R}_{V}+{t}_{V})}^{2}-{R}_{V}^{2}}\cdot {R}_{V}+\frac{(\frac{1+{\nu }_{V}}{{E}_{V}})({R}_{V}^{2}{({R}_{V}+{t}_{V})}^{2}\cdot {p}_{crit})}{{({R}_{V}+{t}_{V})}^{2}-{R}_{V}^{2}}\cdot \frac{1}{{R}_{V}}$$

We assume that the significantly higher hoop stiffness of the carbon fiber cylinder results in a constant diameter of the structure before buckling, making the resulting contact pressures solely dependant on the vessel stiffness, *E*_*V*_, *ν*_*V*_ and *t*_*V*_, as well as the chosen oversizing $\Omega$. Varying $\Omega$ and *E*_*V*_, we obtain Fig. 5c. It shall be noted, that the amplitude of induced imperfections does not vary with different $\Omega$, resulting in a slight spread of solutions of the parameter study, especially towards larger values of $\Psi$. Although the method cannot predict the full deployment process, it estimates the required minimum structural stiffness for a desired deployment environment. Given representative moduli found in the aortic crown^12^, we find that oversizing of up to 3% will lead to a successful deployment of the developed prototype (Ψ ≈ 0.78‰). The maximum contact pressures achievable in this case (up to 1 bar for the largest feasible oversizing), exceeds values reported for successful aortic stent placement of approximately 0.16 bar^30^ achieved with state-of-the-art devices made from super-elastic Nickel-Titanium alloys.

## Concluding Remarks

We have introduced a fully novel design principle, which relies on the introduction of controlled instability patterns in stiff polymer composites. This lead to the development of ultra-thin continuous cylindrical shells that can dilate up to several times their packaged diameter in a purely elastic regime. Although based on stiff and brittle material, we have realized a deploying continuous shell, which was validated considering stringent mechanical boundary conditions of TAVR implants. This includes packaging efficiencies of over 150%, expansion in a confined elastic vessel as well as sufficient and tunable fixation forces inside these vessels. The proposed concept opens up the possibility to realize expandable structures from a whole new set of materials and has great potential to revolutionize the field of TAVR devices and other implants. Advantages are the simple geometry and polymeric nature of the structure, which facilitates the integration of heart valve leaflets thereby increasing durability and fulfilling the requirements for an automated fabrication and assembly.

The presented design principle is versatile and can be applied to realize superior solutions in diverse fields of medicine, which could profit from largely expandable and stiff devices including for instance medication delivery^31^ where the polymeric and cylindrical structures offer large contact areas for medication^32^. The further development of such implants would include the realization of structures from bio-compatible matrix systems like Polyether ether ketone (PEEK), which are already used in prosthetic leaflets in mechanical aortic valve replacements showing great biocompatibility and additionally show similar stiffness and higher toughness to thermoset solutions. Reliable ways to obtain ultra-thin plies of fiber reinforced PEEK however have yet to be found. Furthermore, efficient ways to accurately deliver and deploy the structures need to be found, requiring an advanced delivery and catheter system. Already, the folded structures profile is low enough for transapical TAVI-approaches^13^.

Being independent of structural scale, the concept also enables applications in other fields ranging up to the scale of large expandable frames stiffening structures for deep space exploration^33^. The stiff and highly expandable concept can additionally be exploited for crash or energy-absorbing elements instantly deploying before impact. Furthermore, it should be possible to hierarchically or periodically connect the folded structures increasing the capability of voluminous expansion of the stiff devices.

## Supplementary information

Supplementary Information

Supplementary Movie 1

## Data Availability

The datasets generated during and/or analysed during the current study are available from the corresponding author on reasonable request.
