# Supplementary material for: Stiff Composite Cylinders for Extremely Expandable Structures
Source: Sci Rep. 2019 Nov 4;9:15955. doi: 10.1038/s41598-019-51529-7 (PMC6828719; doi:10.1038/s41598-019-51529-7)
Supplement: Supplementary file 1 — Supplementary Information [file 41598_2019_51529_MOESM1_ESM.pdf]

Supplementary Information for

Stiff Composite Cylinders for Extremely Expandable Structures

Arthur Schlothauer and Paolo Ermanni

Department of Mechanical and Process Engineering
Laboratory of Composite Materials and Adaptive Structures, ETH Zürich
Leonhardstrasse 21, 8092 Zürich, Switzerland

Corresponding Author:
Paolo Ermanni
Email: permanni@ethz.ch

Folding stiff composite cylinders into instability-inspired folding patterns

Although the structure is manufactured from carbon fiber reinforced polymers, its low thickness allows it to be folded by hand. However, at such low scale, an apparatus was needed to fold the structure without violating the minimum bending radii requirements. Since the folding process is mostly in the post-buckling regime of the composite ring, 3D-printable PLA rigs (Figure S1) are sufficient to carry the reaction forces during folding. The second folding step follows a circle involute path winding the lobes around the radius R_2 (s describing the parametrization variable).

$$\begin{bmatrix} x \\ y \end{bmatrix} = \begin{bmatrix} R_2 \cdot (\cos(s) + s \cdot \sin(s)) \\ R_2 \cdot (\sin(s) + s \cdot \cos(s)) \end{bmatrix} \quad (1)$$

The folding rig can be seen in Figure S1. Once pinched by hand and inserted into the 3D-printed pins, the metallic cylinders are rotated around the eccentric axis following the path of the baseplate until they are fully enclosed by the crimped radius R_c. The cylindrical PLA-sleeve can then be strapped around the composite. The rig can then be pulled out from the inside of the PLAcylinder until the structure is free from internal constraints and equilibrates.

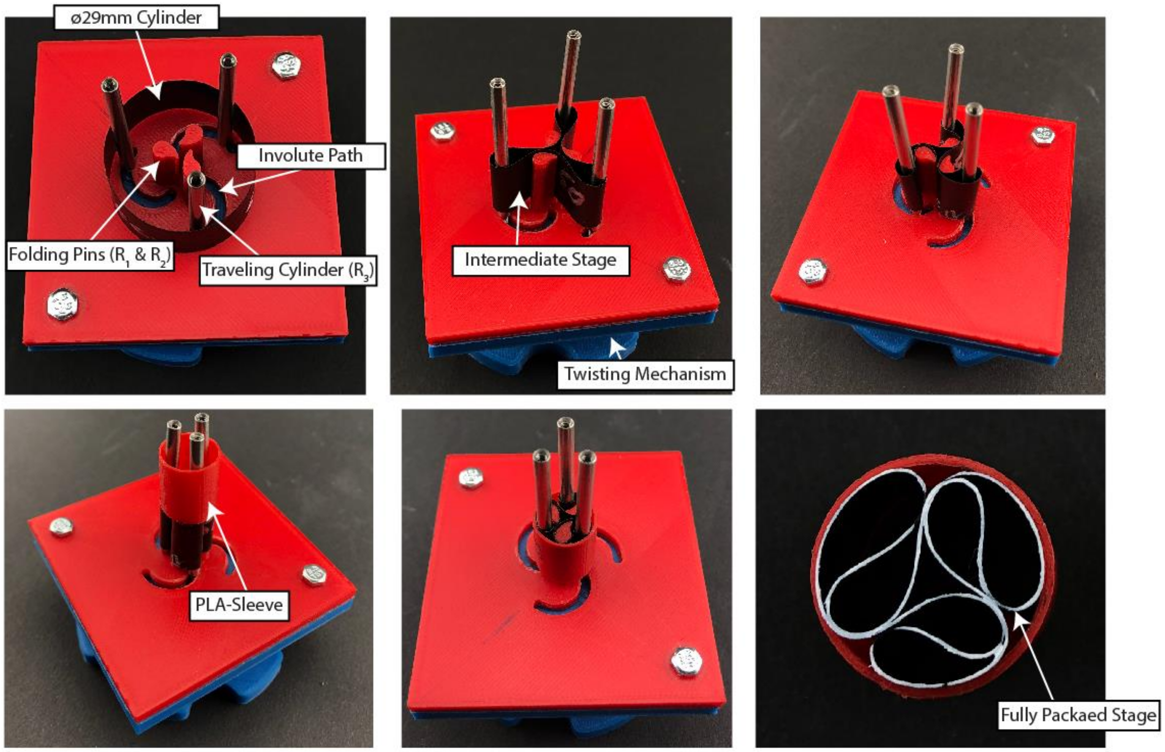

Fig. S1. Folding setup for a foldable composite cylinder with N=3. The PLA folding rig induces the desired folding radii on the structure and prevents overloading.

Derivation of the geometric packaging efficiency of folded cylindrical shells

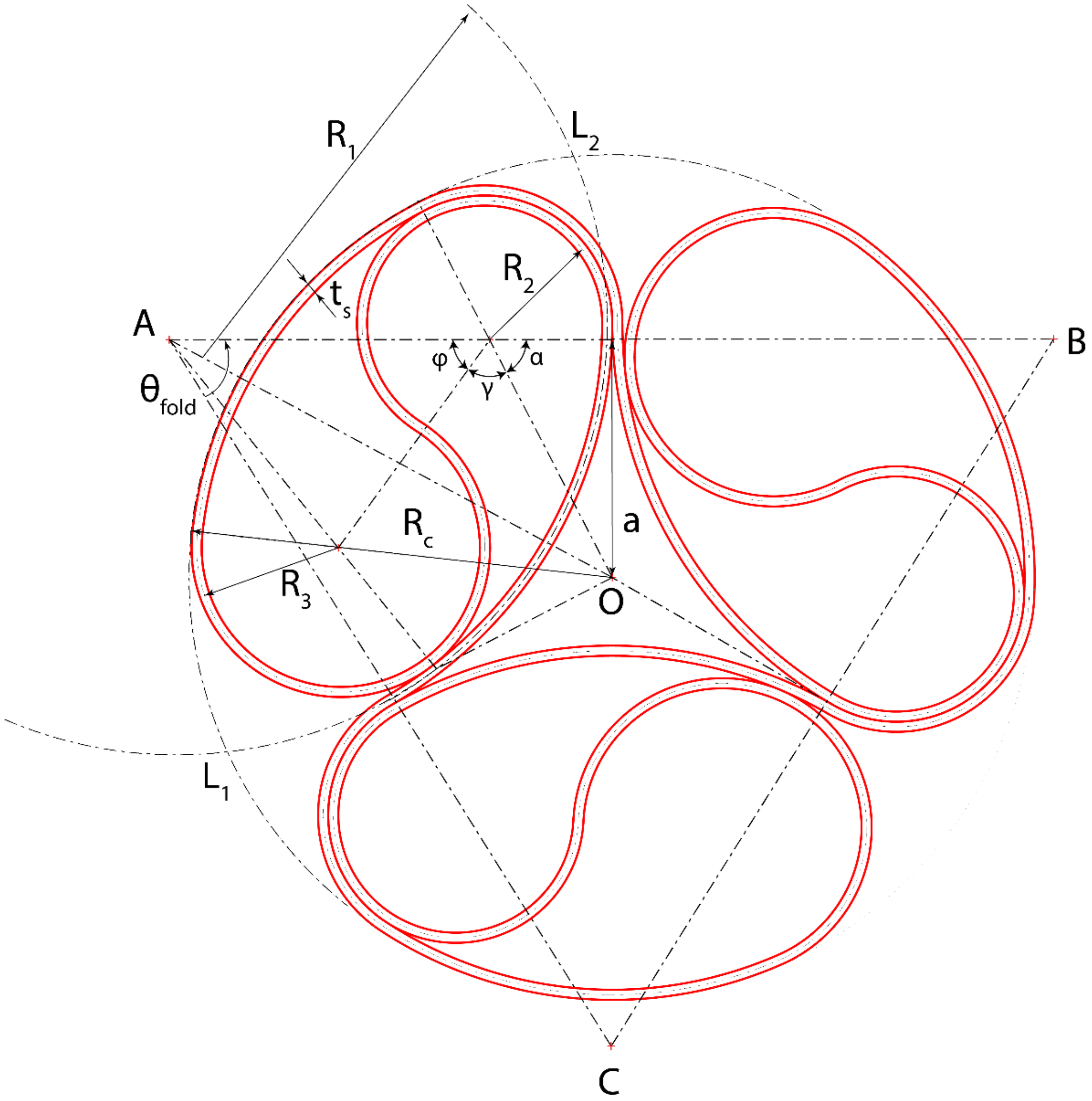

Fig. S2. Trigonometric relations of the packaged structure with non-zero thickness. All radii are now defined with respect to the neutral axis of the shell. Only R_c describes the outer radius of the packaged structure.

The geometrical model for the prediction of optimal packaging efficiency is based on a circle-packaging problem with several tangency requirements (Figure S2). For the simpler case of a structure with t_S=0 with given continuity of the structure, the fully packaged state can be assembled from circular arcs of radii R_1,R_2 and R_3, each of them tangent to its adjacent radius and enclosed by the sleeve radius R_c. We obtain the maximum packaging efficiency by maximizing the arc length of the structure packaged to a given radius, R_c. Therefore we maximize the area of the lens created by the arc of R_1 and R_2 by differentiating it with respect to the radius R_1.

$$\frac{dA_{lens}}{dR_1} = \frac{D(R_1 \cdot \text{acos}(\frac{D^2 + R_1^2 - R_C^2}{2D \cdot R_1}) + R_C^2 \cdot \text{acos}(\frac{D^2 + R_C^2 - R_1^2}{2D \cdot R_C}) - \frac{1}{2}\zeta}{dR_1} = 0 \tag{2}$$

and,

$$\zeta = \sqrt{(-D + R_1 + R_C) \cdot (D + R_1 - R_C) \cdot (D - R_1 + R_C) \cdot (D + R_1 + R_C)} \tag{3}$$

with,

$$D = \sqrt{(a \cdot (2R_1 + t))^2 + (r_1 \cdot \frac{t_S}{2})^2} \tag{4}$$

and a describing the apothem of a regular polygon, which contains a number of sides N (equivalent to the number of lobes of the folded structure),

$$a = \frac{1}{2 \cdot tan(\frac{\pi}{N})}. \tag{5}$$

With $t_S = 0$, maximization of the area yields $R_1 = R_C$. The two smaller radii inside the (now symmetrical) lens are $R_2 = R_3$ given the tangency requirements at their contact point as well as to their confining lens. The value can be derived based on the chosen number of lobes N and correlates with the crimped radius R_C,

$$R_2 = R_3 = R_C \cdot \frac{\sin\left(\frac{\pi}{2N}\right)}{1 + \sin\left(\frac{\pi}{2N}\right)} \tag{6}$$

Given all radii that assemble the geometric representation, the structure is fully defined yielding the overall arc-length which directly results in the unpackaged diameter and hence the packaging efficiency.

For $t_S > 0$ we can follow Equations 2-5 to also derive the packaging efficiency for non-zero thickness structures. The symmetry regarding the radii R_2 and R_3 can not be maintained resulting from a now asymmetric lens L1-L2 (Figure S2). The asymmetry results from the increased thickness at the contact points. Nevertheless we can use the area maximization of the asymmetric lens to obtain the radius R_1, which, using this approach, is not dependent of R_2 and R_3. Tangency requirements are still valid at contact points and between $R_2$and R_3, which are now shifted by $t_S/2$ or $3t_S/2$ respectively. Assuming perfect contact between pinching bodies R_1 and a tightly compressed shell ($2t_S$), we can derive the optimal radius R_2 using the apothem of the polygon and the Pythagorean Theorem:

$$\sqrt{\frac{(a(2R_1 + t_s))^2 + (R_2 + \frac{t_s}{2})^2}{R_c - 3\frac{t_s}{2} - R_2}} - 1 = 0. \tag{7}$$

When solved for $R_{2,}$ we can use the condition that the sum of the angles α, γ and φ needs to be π to create the regular polygon with N sides and internal angle θ_{fold} resulting from the circle packaging

of the first folding stage. The angles can be calculated using the law of cosine between tangent circles, allowing to solve the resulting equation

$$\gamma(R_c, R_2, R_3, t_s) + \alpha(R_c, R_2, t_s) + \phi(R_c, R_2, R_3, t_s) - \pi = 0 \quad (8)$$

numerically for R_3 using MATLAB. For a prescribed ratio of $\tau = \frac{t_s}{R_c}$ and a given lobe count N, this results in the desired folding radii as well as the resulting geometric packaging efficiency of the structure.

Derivation of non-dimensional parameters for deployment

Based on the assumption that the bending stiffness of the confinement is small and the structures bending stiffness significantly lower than its hoop stiffness, we identify the ratio between critical buckling pressure of the deploying structure and hoop stiffness of the constraint as driving factor for deployment. This ratio can be split into two non-dimensional parameters that allow to decouple conveniently tunable geometrical parameters (Oversizing Ω) from stiffness driven parameters (Ψ). For a perfect cylindrical composite we can calculate the critical buckling pressure given its layup and radius [1] and the vessel hoop stiffness is approximated using thin shell theory. It shall be noted that for radius to thickness values of human arteries, thin shell theory is drastically simplified, however it enables easier comparison and understanding of the relations during the deployment process.

$$\frac{p_{crit,S}}{E_{hoop,V}} = \frac{D_{22} \cdot R_V}{E_V \cdot t_V \cdot R_0^3} = \frac{D_{22}}{E_V \cdot t_V \cdot R_0^2} \cdot \frac{1}{\Omega + 1} = \Psi \cdot \frac{1}{\Omega + 1}. \quad (9)$$

Deployment Test Setup

Figure S3 shows the deployment test set-up of the deployment test. All fixtures where printed from PLA. The silicone sleeve was manufactured by casting Altropol Protosil RTV 245 Silicone in 3D printed molds. Therefore, the length was chosen to be at least 4x larger than the structure length so edge effects of the clamping do not influence the deployment. The silicone was circumferentially glued onto PLA vessel fixations (red in Figure S3) which then where screwed into the PLA fixtures. The center axis of the camera was aligned with the silicone vessel. Simultaneous release of the structure was achieved by pushing the structure out of its constraint.

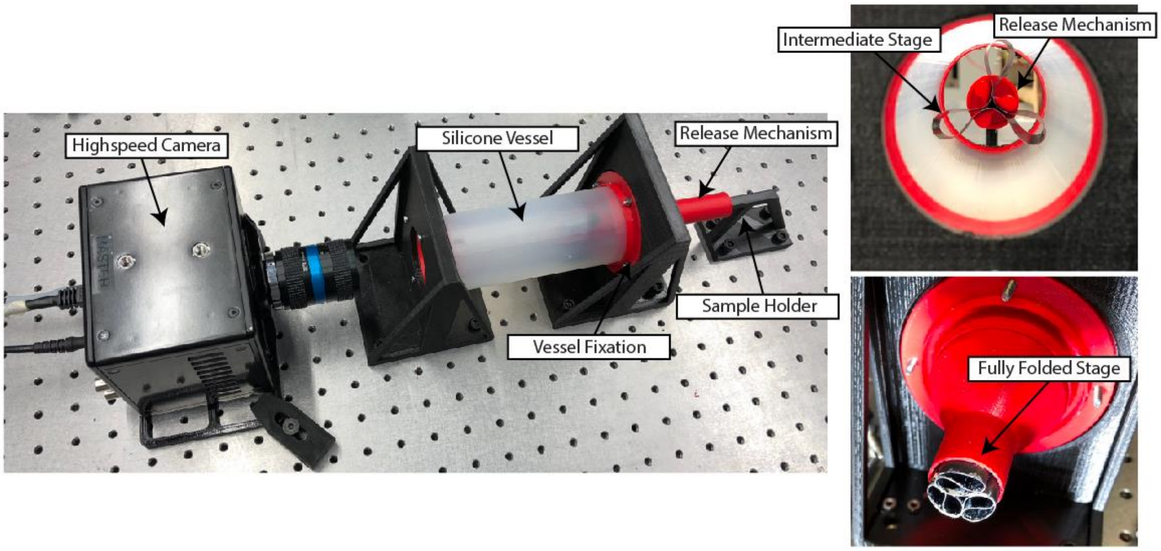

Fig. S3. Deployment test set-up for the proof of concept of the expandable devices.

Movie S1. Supplementary Movie S1 shows the recording of a deployment process of a fully folded structure which is released from its sleeve seen in Figure S3. The high-speed camera recorded with 8500FPS. The edges of the structure were painted in white shortly before the experiment to facilitate contrast. The white particles in the video show excessive white ink accelerated by the inertial forces during deployment.

References

1. Rasheed, H. A. and Yousif, O. H., Buckling of Thin Laminated Orthotropic Composite Rings/Long Cylinders Under External Pressure," International Journal of Structural Stability and Dynamics 1(4), 485-507 (2001).
